# Diagnosis of Dengue Infection Using Conventional and Biosensor Based Techniques

**DOI:** 10.3390/v7102877

**Published:** 2015-10-19

**Authors:** Om Parkash, Rafidah Hanim Shueb

**Affiliations:** Department of Medical Microbiology and Parasitology, School of Medical Science, Universiti Sains Malaysia, 16150 Kubang Kerian, Kelantan, Malaysia; hanimshueb@gmail.com

**Keywords:** dengue, diagnosis, detection, biosensor, rt-PCR, dengue specific IgM, NS1, viral isolation

## Abstract

Dengue is an arthropod-borne viral disease caused by four antigenically different serotypes of dengue virus. This disease is considered as a major public health concern around the world. Currently, there is no licensed vaccine or antiviral drug available for the prevention and treatment of dengue disease. Moreover, clinical features of dengue are indistinguishable from other infectious diseases such as malaria, chikungunya, rickettsia and leptospira. Therefore, prompt and accurate laboratory diagnostic test is urgently required for disease confirmation and patient triage. The traditional diagnostic techniques for the dengue virus are viral detection in cell culture, serological testing, and RNA amplification using reverse transcriptase PCR. This paper discusses the conventional laboratory methods used for the diagnosis of dengue during the acute and convalescent phase and highlights the advantages and limitations of these routine laboratory tests. Subsequently, the biosensor based assays developed using various transducers for the detection of dengue are also reviewed.

## 1. Introduction

Dengue, a vector-borne disease, is prevalent in tropical and sub-tropical regions of the world and associated with endemic as well as in epidemic transmission cycles. Dengue usually causes inapparent infection, but it could also lead to other wide range of clinical symptoms/diseases including mild dengue fever and severe dengue [1]. This disease is caused by dengue virus (DENV), which is a member of the *Flavivirus* genus within the *Flaviviridae* family. This virus is classified into four antigenically related but genetically distinctive serotypes, DENV-1, -2, -3 and DENV-4. The four DENV serotypes differ in the nucleotide sequence by 25–35 base pairs and each serotype is capable of causing dengue. Out of these four different serotypes, DENV-4 appears to be the most divergent serotype followed by DENV-2 while DENV-1 and DENV-3 are more closely related [2]. Infection with any serotype provides long-term immunity to that specific serotype only, but limited and temporary immunity to the other three serotypes [3,4]. Epidemiological studies have shown that secondary infection with different serotypes may lead to more severe dengue [5].

DENV is surrounded by an envelope which encloses single-stranded positive sense RNA comprising approximately 1100 nucleotide base. Translation of viral RNA produces a single polypeptide, which upon the proteolytic cleavage by proteases results in the formation of three structural proteins (capsid, membrane and envelope) and seven non-structural (NS) proteins (NS1, NS2a, NS2b, NS3, NS4a, NS4b, NS5) [6,7]. The structural proteins form the coat of the virus and help in delivering the RNA to target host cell. The non-structural proteins organize the production of a new virus in the host cell [8]. Dengue is spread between people by the mosquitoes *Aedes aegypti* and *Aedes albopictus*, through a human-to-mosquito-to-human cycle of transmission [9]. DENV enters into the blood through a mosquito bite and infects the peripheral blood mononuclear cells. Clinical symptoms typically appear 4–7 days following mosquito bite and may persist for 3–10 days [10].

## 2. Dengue as a Global Health Concern

Dengue is a serious health issue in tropical and subtropical countries around the world. Currently, the disease is endemic in more than 100 countries and imperils more than half of the world population. Three hundred and ninety million dengue infections are estimated to occur every year, and of that, 96 million are serious enough for patients to seek medical attention [1,11,12]. The dengue case burden and the number of countries reporting outbreaks have increased 10-fold in the last 30 years [13]. This disease is now endemic in Africa, Americas, the Eastern Mediterranean, Southeast Asia and the Western Pacific. Southeast Asia and the Western Pacific are the most seriously affected areas with the former region contributes 52% cases annually [6,14,15]. Consequently, dengue inflicts a significant health, economic and social burden on the populations of endemic areas. A recent survey conducted in 12 countries within the South East Asia region revealed an annual economic burden of nearly 1 billion dollar incurred due to dengue [16].

## 3. Causes for Emergence of Dengue

There are several factors associated with the current increase of dengue incidence including uncontrolled growth of urban population, rapid urbanization, lack of vector control in dengue endemic areas, increased air travel, and inadequate public health care systems such as sewage and waste management [17,18]. Other important factors include temperature, humidity and rainfall [19].

## 4. A Brief History of Dengue

The word “dengue” comes from the Swahili language, which means a disease caused by an evil spirit. Although dengue-like disease was reported from China as far back as 992 A.D, the first major dengue epidemics were only reported in the late 17th century [20]. The occurrence of dengue as an epidemic was not frequent at that time. Nonetheless, high incidence of severe dengue was demonstrated in the South-East Asia region after World War II, as urbanization provided a suitable condition for virus propagation [21]. In the South-East Asia, outbreaks of dengue fever were initially reported in 1954 and 1956 in Manila, Philippines, and in 1958 in Bangkok [22]. The first epidemic in Singapore was recorded in 1960 with a low mortality rate [23]. During the 1960s and 1970s, the disease caused outbreaks in India, Malaysia, Singapore, Vietnam, Indonesia and Myanmar. At present, dengue has spread west into Pakistan, Sri Lanka, Maldives and east into Taiwan and China [22].

## 5. Laboratory Diagnosis and Its Significance

**Figure 1:**
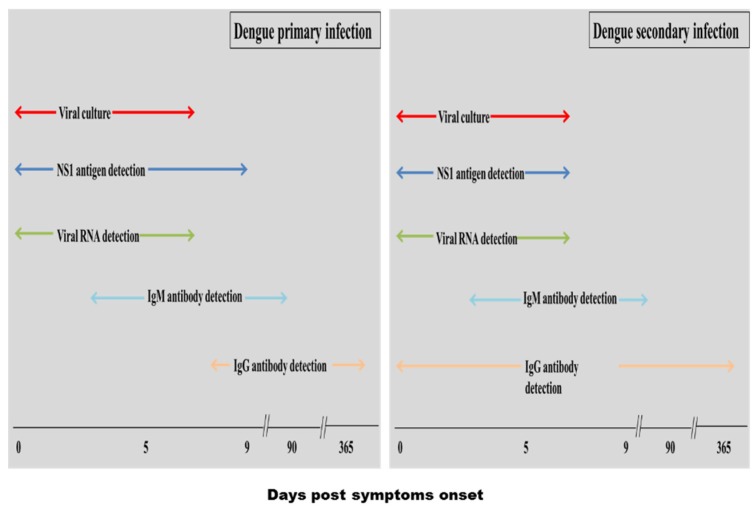
Suitability of dengue diagnostics at different phases of illness.

Clinical features of dengue infection, during acute and convalescent, are quite non-specific and mimic many other diseases (Table 1). Thus, could be easily misinterpreted. Moreover, there is no specific treatment available for this disease. Thus, accurate laboratory diagnosis is very helpful in controlling this disease [12]. An early diagnosis helps in prompt patient management and immediate implementation of appropriate vector control measures which in turn helps to prevent the spread of the infection. Additionally, diagnostics provide key data on the epidemiology and health burden of dengue, which is very useful for accurate public health surveillance [24]. Currently, many in-house assays or rapid diagnostic tests (RDTs) have been developed are commercially available and used in routine diagnostic laboratories. However, selection of a proper test is dependent on various factors such as viremia period and infection status (primary or secondary) (Figure 1).

**Table 1:** Differential diagnosis for dengue illness.

Clinical symptoms	Differential diagnoses
Flu-like	Influenza, Measles, Chikungunya, Adenovirus infection, Infectious mononucleosis, Acute HIV seroconversion illness
Rash	Rubella, Zika fever, West Nile fever, Measles, Scarlet fever, Meningococcal infection, Chikungunya, Drug
Diarrhoea	Rotavirus infection, Norovirus, Cytomegalovirus, Viral hepatitis, Food poisoning
Muscle/Joint pain	West Nile fever, Zika fever, Chikungunya
Neurological manifestation	Meningoencephalitis, Tick-borne encephalitis, West Nile fever, Japanese encephalitis, Febrile seizures
Bleeding tendency	Yellow fever, Ebola, Marburg
Thrombocytopenia	Rubella, Epstein-Barr virus, Parvovirus

### 5.1. Conventional Diagnosis Methods

Several conventional laboratory techniques/tools are available for the diagnosis of dengue. Confirmation of dengue could be made through virus isolation, genome amplification as well as antigen and antibody detection via serology.

#### 5.1.1. Viral Culture

Virus isolation has always been considered as the gold standard for the diagnosis of dengue infection [25]. For DENV cultivation, three methods have been reported, including inoculation of specimens into mosquitoes, various *in vitro* cultured cell lines, and intracerebrally in mice. Usually, common specimens including plasma, serum, peripheral blood, cerebrospinal fluid, pleural fluid and immune system tissues such as the liver, spleen and lymph node are used for virus culture. After the cultivation of the specimen, a confirmation assay that includes immunofluorescence assay or reverse transcriptase polymerase chain reaction (RT-PCR) is performed once cytopathic effect in infected cells is observed. Although virus isolation method provides high specificity, various practical aspects limit its use. First, this technique is tedious and requires long incubation time (7–12 days) for virus cultivation and confirmation [26]. Second, it requires appropriate lab facilities with well-trained personnel. Third, low level of virus titre in serum or blood is not suitable for virus culture [11]. Lastly, the optimal window for culturing the virus is limited to 0–7 days following the onset of symptoms, as DENV is detectable only during the acute phase of infection, prior to development of dengue specific antibody response [27,28]. Therefore, despite being the gold standard for identification of dengue infection, this approach is not practical in routine diagnostic laboratories.

#### 5.1.2. Nucleic Acid Amplification

Detection of DENV by nucleic acid amplification using RT-PCR is suggestive of an acute infection [29]. This technique provides several advantages including ability to differentiate DENV serotypes, can be a quantitative assay and has higher sensitivity when combined with real-time technology [29]. However, the RT-PCR test is expensive and requires specialized equipment and well-trained personnel, thus, limiting its use in many developing countries [30,31,32,33].

#### 5.1.3. Serological Diagnosis

Commercial serological assays are commonly used in diagnostic laboratories for dengue confirmations. Serological assays are comparatively simple to perform and the specimens required for the assay, such as serum or plasma, are stable in the tropical climate. Consequently, these techniques can be used in various settings such as surveillance, health care facilities and travel clinics. However, the applicability of serological tests in dengue endemic areas should be evaluated against the potential cross-reactivity with other circulating flaviviruses [34].

Serological tests are more widely used for the detection of dengue infections in resources limited countries as they are relatively inexpensive and easy to handle. These tests include hemagglutination inhibition (HI) assay and enzyme-linked immunosorbent assay (ELISA) to detect immunoglobulin M (IgM) and immunoglobulin G (IgG) antibodies. RDTs for some of these tests are also available commercially.

##### HI Assay

This test used to be very popular and acted as the gold standard for the diagnosis of dengue. The principle of this test is based on the ability of dengue envelope protein to agglutinate red blood cells. Anti-dengue antibodies present in dengue-infected patient sera inhibit this agglutination and the extent of this inhibition is measured in HI test [35]. HI antibodies last for long periods, and thus, this test is very useful for seroepidemiologic studies [36]. The HI test can be used to detect and differentiate between primary (gradual increase to moderate titer of antibody) and secondary (rapid increase to high titer) DENV infections due to its simplicity and sensitivity [28,37]. HI antibody titers during primary infections peak at 1:640 whereas titers of 1:1280 or greater are common during secondary infections [38]. The test was commercially developed into a simple, rapid, sensitive and specific serological kit. However, the test was eventually replaced by ELISA based method for the detection of dengue specific IgM and IgG antibodies due to its numerous practical limitations [39,40]. Firstly, it cannot provide prompt diagnosis since paired sera are required. Secondly, HI test also exhibits high cross-reactivity, resulting in impractical application in countries where flavivirus infections are endemic. Lastly, it requires chemical pre-treatment to remove nonspecific inhibitors of haemagglutination [36,37,41,42].

##### Detection of Immunoglobulin M (IgM) Antibody

The detection of dengue-specific IgM antibody is a useful diagnostic tool in resource limited countries, particularly after a very short viremia period [38]. During primary DENV infection, IgM antibody is the first immunoglobulin isotype to appear while IgG antibody appears after a few days of IgM appearance. In contrast, during secondary DENV infection, IgG antibody is the first to appear after onset of symptoms while IgM antibodies appear after a few days at low titer or even undetectable in some patients [28]. Therefore, the presence of IgG and low levels of IgM during secondary infection often obstruct accurate diagnosis when direct IgM ELISA is used. Consequently, dengue IgM capture assays (MAC-ELISA) were designed to overcome the antigen-binding competition between IgG and IgM and MAC-ELISA has now become a widely used method for the detection of anti-DENV IgM antibodies [43]. This method shows a sensitivity and specificity of 90% and 98%, respectively, in samples collected after seroconversion [44,45].

RDTs based on lateral flow and particle agglutination format that provides result as early as 15 min have also been developed. However, the sensitivity range of RDTs in comparison to ELISA suggest that it is less sensitive test (Table 2). Although due to the advent of technology, newer generation of RDTs and ELISA may have higher sensitivity, the major drawbacks of all IgM antibody-based assays are that they can cross-react with other flaviviruses and they may provide inaccurate results if patients had recent dengue infection. 

**Table 2:** Sensitivity and specificity of evaluated commercialized dengue specific IgM ELISA and rapid test.

Test format	Brand	Assay time	Sensitivity	Specificity	Ref.
ELISA	Dengue Fever Virus IgM Capture, Focus Diagnostics	225	98.6	79.9	[46]
	Pathozyme M Dengue, Omega	120	61.5	84.6	[46]
	Pathozyme M Dengue Capture, Omega	110	83.5	86.5	[45]
	Dengue IgM Capture, Panbio	130	89.5 87.6	861 88.1	[45,47]
	SD Dengue IgM Capture, Standard Diagnostics	130	84.9	97.3	[47]
	InBios IgM ELISA	90	88.7	93.1	[48]
Rapid test	Panbio Dengue Duo Cassette (IgM/IgG)	15	77.8 92.1	90.6 62.2	[46,49]
	Hapalyse dengue-M PA kit, Pentax	90	97.7	76.6	[46]
	SD Bioline Dengue IgG/IgM	15–20	60.9 87.3%	90 86.8	[46,49]
	Dengue check WB, Zephyr (IgM/IgG)	15	20.5	86.7	[46]

##### Detection of IgG Antibody

Dengue specific IgG ELISA is introduced as an alternative to HI assay. The dengue IgG capture ELISA (GAC-ELISA) is the most commonly employed format, in addition to indirect IgG ELISA detection method. The IgG ELISA assay uses similar polyvalent viral antigen as the MAC-ELISA and has a good correlation with HI assay. Meanwhile, IgG avidity test can be used to classify between primary and a secondary dengue infection in patient sera. This avidity test is based on the principle that the first antibodies produced after primary infection exhibit low avidity (binding affinity) to an antigen than those produced later [38,50,51]. The IgG ELISA offers several advantages such as rapid, easy to perform and is suitable for large-scale surveillance studies [52]. Moreover, IgG based assay shows higher sensitivity than HI assay. However, similar to the HI test, the IgG-based ELISA assay displays the same broad cross-reactivity with other circulating flaviviruses. Additionally, this serological method could not identify the infecting DENV serotype [38].

##### Detection of DENV NS1 Antigen

DENV NS1 is a highly conserved glycoprotein, expressed as both membrane-associated and secreted forms. Secreted NS1 has been detected ranging from 2–0.04 µg·mL^−1^ in the serum of dengue-infected patients during the early stages of the disease [53,54,55]. A high NS1 level has been demonstrated to circulate as early as one day after onset of symptoms up to early convalescences, thus provides an alternative to virus culture or PCR for early dengue diagnosis when IgM or IgG antibodies are not present yet in dengue infected patients [56].

Circulating dengue NS1 in sera can be detected either using ELISA assay or lateral flow based RDTs. Several kits manufactured by various companies are available in the market although sensitivity and specificity vary between them. A number of studies have evaluated the sensitivity and specificity of these commercially available dengue NS1 ELISA and RDTs (Table 3) [56].

**Table 3:** Sensitivity and specificity of evaluated commercialized NS1 ELISA and rapid test.

Test format	Brand	Assay time (Min)	Sensitivity %	Specificity %	Ref.
ELISA	Platelia Dengue NS1 Ag Kit, Biorad	140	89.4 83.6	97.4 98.7	[26,57]
	DENV Detect NS1 ELISA, Inbios	111	95.9	100	[57]
	Pan-E Dengue Early ELISA, Panbio	160	85.5 72.3	95.5 100	[26,57]
	SD Dengue NS1 Ag ELISA	160	76.7	98.31	[58]
Rapid test	Dengue NS1 Detect , Inbios	30	86.0 76.5	100 97.4	[57,59]
	Biorad NS1 Ag Strip	15–30	72.8 79.1	100 100	[57,59]
	Panbio NS1 Ag Strip	15	71.9	95	[57]
	SD Dengue Duo	15–20	70.6 72.4	100 73.4	[26,57]

### 5.2. Biosensor Based Methods for Dengue

Detection of IgM and NS1 based on RDTs and ELISA methods are the most widely used dengue assays in many countries. Although ELISA based assay have been shown to be more sensitive than RDTs, the former test lacks portability and thus, preventing its use in private clinics and field work. Because of this, many researchers have delved into biosensors as alternative new technology for the detection of DENV and dengue antibodies since this technique has several advantages such as higher sensitivity, cost-effective, simple fabrication, possible miniaturization, rapid outcome with quantitative analysis and possible on-site monitoring [60]. The most well-known commercially available biosensors are the glucose sensor and home pregnancy test [61]. However, unlike RDTs and ELISA based assay, development of biosensor for dengue diagnosis is still in its infancy stage and has not been commercialised. Based on different types of transducer, this section discusses three main types of biosensor, electrochemical, piezoelectric and optical, which have been developed for the diagnosis of dengue infection.

#### 5.2.1. Electrochemical Biosensor

**Figure 2:**
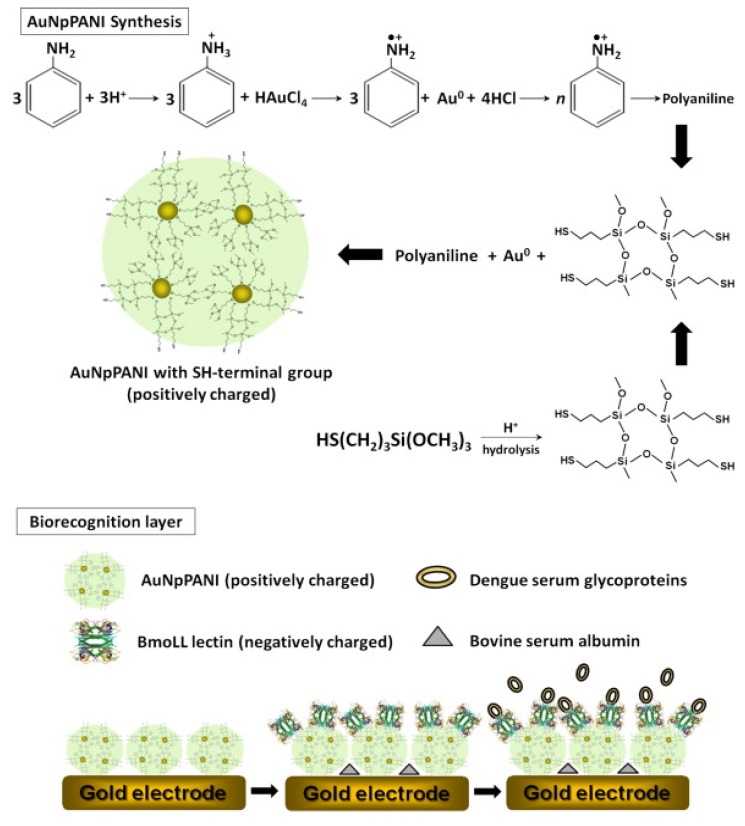
Mechanism for the reaction of aniline with chloroauric acid trihydrate and schematic representation of the AuNpPANI-BmoLL-BSA-DEN biosensor system (Retrieved from [65]).

Electrochemical biosensors monitor the measurable current signals generated following electrochemical oxidation and reduction reactions. The current signals recorded during the reaction resulting from the recognition of a target analyte and measurement of signals is proportional to target analyte concentrations [62]. Among the reported electrochemical biosensor was lectin based electrochemical impedance spectroscopy analysis developed to discriminate serum glycoproteins in dengue-infected patients. The lectin was introduced as an alternative to a monoclonal antibody to detect dengue serum glycoprotein (SG). Lectins are carbohydrate-binding proteins that are highly specific for carbohydrate structure. In one study, concanavalin A (Con A) lectin, a type of lectin specific to Gly/Man lectin and can be used for the recognition of serum glycoprotein, was employed. This lectin was immobilized on the gold electrodes through gold nanoparticles (AuNps) and polyvinyl butyral (PVB). Electrochemical impedance spectroscopy (EIS) was then used to measure the electrochemical response. It was demonstrated that the biosensor showed different interaction to serum glycoprotein and serum without glycoprotein. The electrodes modified with serum glycoprotein demonstrated higher electron transfer resistances than those modified with BSA, indicating an agglutination reaction on the electrodes. The resulting biosensor detected dengue serum glycoproteins from dengue-infected patients with a detection limit of 80 dilution fold [63]. In a similar type of study, the serum glycoproteins were detected by a different type of lectin, *i.e.*, Cratylia mollis (CramoLL). However, in this study, a slightly different immobilization method for the lectin was used; CramoLL was immobilized on Fe_3_O_4_ modified gold nanoparticles (AuNps). Notably, this study dealt with three different serotypes of dengue and the developed biosensor showed a wide linear response to various concentrations of dengue serotypes 1, 2 and 3 in sera [64]. Another study had used bauhinia monandra (BmoLL), another different type of lectin, to detect DENV. Immobilization of this lectin was carried out on a novel gold nanoparticles-polyaniline hybrid composite (AuNPPANI) (Figure 2). The particular biosensor was able to detect and differentiate three dengue serotypes as the viruses have higher electron-transfer resistance than the negative samples, indicating that lectin-glycoprotein complex behaves as the unreactive electron and mass-transfer blocking layer, and prevents the diffusion of ferricyanide toward the electron surface. This lectin-based sensor showed better sensitivity for the detection of dengue serum glycoproteins than a previous study [63] with a detection limit of 1:150 [65]. 

Apart from lectin, some other studies have employed specific dengue antibody to detect dengue serum glycoprotein. A label-free electrochemical immunosensor was developed on gold electrode, acquired from a recordable compact disk (CD-trode), for the detection NS1 antigen [66]. Protein A was used for the oriented immobilization of anti-NS1 capture antibody on the gold electrode. Differential pulse voltammetry (DPV) was performed to measure electrochemical response occurred during the interaction between NS1 antigen and anti-NS1 antibody immobilized on the electrode. Interestingly, this biosensor was a quantitative assay and was very sensitive with detection limit of 0.33 ng·mL^−1^ [66].

Some researchers have exploited more advanced technology/method such as nanotechnology and oriented capture antibody immobilization technique to increase the sensitivity of the biosensor. Since nanomaterial provides extraordinary chemical and physical properties such as large surface per unit mass (50–500 m^2^/g) and excellent mechanical and electrical properties, these make them particularly useful for electronic detection of biomolecules. In addition, the surface of nanomaterial can be functionalized with appropriate chemical groups so that the desired biomolecules (nucleic acids, enzymes, carbohydrates) can be immobilized [67]. On the other hand, oriented antibody immobilization method comparatively captures more antigens than random antibody immobilization method, therefore, it is a crucial factor to be considered for improving the sensitivity of the immunoassay. Immobilization of antibodies is considered to be properly oriented and perfectly active when the Fc region, which has no antigen binding affinity, is immobilized on a surface rather than the antigen-binding sites of the antibodies [68]. In this regard, one specific example is the carbon nanotube (CNT) modified screen printed electrode specific to detect dengue NS1 glycoprotein [55]. The prominent feature of this immunosensor was the oriented covalent immobilization of anti-NS1 antibody on working electrode, resulting in a better sensitivity of the assay. A much lower detection limit of 12 ng·mL^−1^ was demonstrated in this study, which is within the NS1 concentration range in dengue infected serum samples. Incorporation of the CNT into carbon ink also proved to be useful and enhances the sensitivity and reproducibility of the immunosensor [55]. Although this immunosensor showed great sensitivity, its practical application is still debatable as it was not tested on real clinical samples.

Recently, some other studies have developed an electrochemical biosensor for the detection of NS1 in real serum samples [69,70,71,72]. An electrochemical biosensor using hybrid membrane composed of ConA and lipid membrane has been developed to detect glycoprotein from dengue-infected samples [69]. Another study has developed similar biosensor but using CramoLL lectin to discriminate glycoproteins from sera of a dengue-infected patient. However, in this study CramoLL lectin was deposited by electrostatic interactions on hybrid nanocomposite composed of gold nanoparticles (AuNps) and polyaniline (PANI) [70]. Streptavidin/biotin based oriented immobilization method has also been applied to electrochemical immunosensor for the detection of dengue serum glycoprotein in real serum samples. In one study, screen printed carbon electrode was pre-treated with streptavidin/biotin system for the immobilization of the anti-dengue NS1 capture antibody [72]. Incorporation of this technique resulted in a well-oriented immobilization of the capture antibody and consequently enhanced the sensitivity of the assay with a detection limit of 0.03 µg·mL^−1^. In terms of versatility, egg yolk immunoglobulin (IgY) has been employed as an alternative biorecognition component to conventional mammalian antibodies for the detection of DENV NS1 protein [71]. Although, the assay demonstrated a detection limit of 0.09 µg·mL^−1^ which were higher than the previous study reported by Dias and co-worker [55], this assay was label free. As mentioned above, NS1 levels vary from 0.04 μg·mL^−1^ to 2 μg·mL^−1^ in serum samples of dengue patients [55]. In this context, a number of developed immunosensors have shown excellent sensitivity. For example, Dias and co-workers reported 0.12 ng/mL while Cavalcanti and co-workers reported 0.33ng/mL limit of detection for their immunosensors, which is lower than 7ng/mL and 3.04 ng/mL limit of detection reported in some ELISA-based assays [73,74].

Apart from serum glycoprotein, some biosensors have been developed to target other DENV proteins. A chitosan-modified carbon fiber electrode (CFE) was developed to detect DENV envelope protein. In terms of sensitivity, the immunosensor had a better limit of detection for DENV than previously described studies (0.94 ng·mL^−1^), and a linear range from 1.0 to 175 ng·mL^−1^ which is clinically relevant to dengue diagnosis. This immunosensor presented an inexpensive assay as the CFE is cheaper than other electrodes such as gold or platinum [33]. Similarly, DENV type 2 virus has been detected on an alumina-modified platinum electrode [75]. The main features of this nanobiosensor were short analysis time of 50 min and detection limit of 1 PFU·mL^−1^ with a linear detection range from 1 to 10^3^ PFU·mL^−1^. Additionally, this assay showed no cross-reactivity against other viruses such as chikungunya virus, west nile virus and DENV-3. The reproducibility of the nanobiosensor was acceptable with relative standard deviation (RSD) of 5.9% for triplicate analysis [75]. In a separate but similar type of development, a label-free immunosensor was developed on a small and thin piece of alumina membrane as the sensing platform for the detection of dengue. Comparatively, this alumina membrane based sensor was rapid with short assay time of 40 min and demonstrated good sensitivity with a detection limit of 0.230 PFU·mL^−1^ [76].

**Figure 3:**
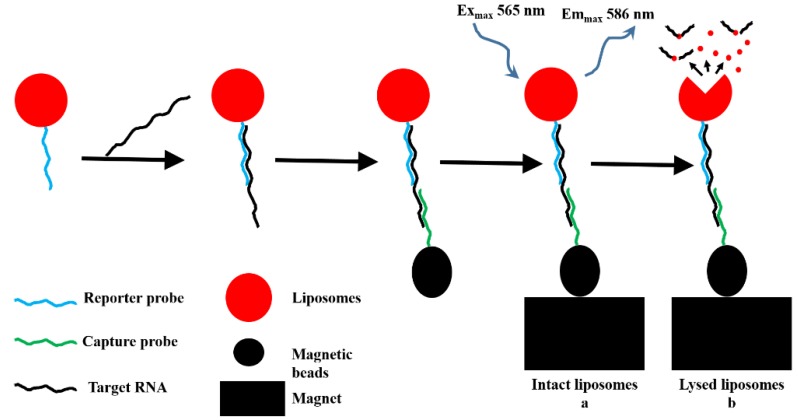
The principle scheme of the biosensor based on DNA/RNA hybridization, magnetic bead complex formation and fluorescence detection of RNA-specific complexes *via* intact (**a**) and lysed (**b**) liposomes (Retrieved from [77]).

Besides electrochemical immunosensor, there are also numerous research on the development of electrochemical genosensor for the identification of dengue nucleic acid. Among the reported genosensor is an electrochemical microfluidic biosensor developed for the quantification of target RNA. Interestingly this study designed an inexpensive miniaturized electrochemical detection system (miniEC) and compared its performance to a standard lab-bench electrochemical workstation. The detection mechanism of this electrochemical sensor was based on the hybridization of target nucleic acid with reporter probe DNA conjugated with liposome which interacts with electrochemically active redox species. Upon hybridization, the liposomes were lysed and resulting electrochemical response were measured with miniEC (Figure 3). In terms of performance, this assay showed ten times lower detection limit than the standard lab-bench electrochemical workstation [77].

In another study, a label-free electrochemical genosensor utilizing pencil graphite electrode was developed for the detection of a specific oligonucleotide sequence of DENV Type 1. Hybridization between the probe and the target oligonucleotides was electrochemically monitored through oxidation of guanine. Important features of this genosensor include that ordinary pencil lead was used during preparation of the pencil graphite electrode and simple immobilization technique of the oligonucleotide probe was utilized. In addition, the assay showed a good sensitivity with a detection limit of 0.92 nM and a very short detection time for hybridized probe and its complementary DNA [78]. Recent developments in genosensor also lead to the detection of particular dengue serotype. In one dengue genosensor, a positively charged gold nanoparticle-polyaniline hybrid composite (AuPANI) was immobilized on gold electrode using its SH-terminal and then negatively charged serotype specific primers were deposited on positively charged AuPANI layer. this assay was highly sensitive as it could discriminate DENV serotypes in the sera of dengue-infected patients at picomolar concentrations [79]. In addition, nanotechnology based ultrasensitive DNA biosensor has been developed using nanoporous alumina membrane to detect DENV serotype 1 and 3 [80]. The advantage of using porous nanoelectrode is the high surface area that increases probe loading which in turn enhances the loading of target analytes, and consequently, increases the sensitivity of the assay. Indeed, it was shown that this biosensor has an ultrasensitive detection limit of 9.55 × 10^−12^ M for the target DNA. Unlike other electrochemical detection methods, where labeled probe and redox species are required for electrochemical signals, this assay only utilizes Fe(CN)_6_^−4^ for DPV based electrochemical signals. The other advantages of this biosensor include its simple preparation and carving of nanostructure which is comparatively easy than other conventional lithography [80]. In a separate study, a genosensor was developed using integrated sensing membrane based on anodic aluminum oxide membrane. The detection mechanism of this study was quite interesting. First the probe DNA was covalently immobilized within the nanopores of the membrane using a cross-linker. Afterward, the target DNA was deposited within the same membrane. The complementary hybridization of the target DNA to its probe within the pore resulted in blocking of nanopores. This blocking of pores caused the impedance changes and provided detection signals. This study demonstrated that the pore resistance was directly proportional to the concentration of DNA. The genosensor exhibited substantial sensitivity with a detection limit of 2.7 × 10^−12^ M target DNA analyte. Additionally, this genosensor was capable of discriminating the complementary target sequence from several mismatch to a single base mismatch sequences [81].

#### 5.2.2. Piezoelectric Biosensor

Piezoelectric biosensor is a mass sensitive detector which utilize mechanically induced changes to produce electrical signals. The transducer used in a piezoelectric biosensor is made of a piezoelectric material such as quartz and the bioreceptor is coated with the piezoelectric material which resonates at a natural frequency. This frequency is controlled by an external potential which produces an oscillating electric field. The biorecognition event causes variation in resonance frequency resulting in current changes which is detected by crystal microbalance (QCM) [62]. Among the reported piezoelectric biosensor is an immunochip for the detection of DENV NS1 and envelope proteins. This immunochip was developed on QCM and two different monoclonal antibodies for the detection of NS1 and DENV envelope protein were immobilized on two separate immunochips. In addition, a cocktail immunochip containing a mixture of both antibodies was also developed. Three different antibody immobilization techniques were investigated and compared in this study, and protein A was demonstrated to be a better immobilization agent than glutaraldehyde and carbodiimide. The sensitivity of this immunochip assay was 100-fold higher than the traditional sandwich ELISA method while assay time was about one hour. Comparatively, cocktail immunochip demonstrated higher sensitivity than the normal immunochip [82]. This cocktail immunochip could simultaneously detect NS1 antigen and DENV which circulate at the same time during the acute phase of dengue infection. Similarly, another group of researcher also has designed a piezoelectric biosensor for detection of DENV envelope protein and NS1 glycoprotein [83]. However, in this study, real serum samples were used for the early diagnosis of dengue. In order to increase the assay sensitivity, various serum pre-treatment methods were investigated and compared. It was found that cibacron blue 3GA gel-heat denature method was the most efficient sample pre-treatment technique with a detection limit of 1.727 µg·mL^−1^ and 0.740 µg·mL^−1^ for E protein and NS1 protein, respectively [83]. A novel biosensing method for piezoelectric biosensor has also been developed. A thin molecularly imprinted polymer specific for nonstructural protein 1, formed by polymerization of monomers, was coated on QCM. These resulting artificial receptors on QCM form amino-acid like cavities that can discriminate epitopes on dengue NS1 glycoprotein. This immunochip was shown to be able to detect all four DENV serotypes during the acute phase of dengue infection with a detection limit ranged from 1–10 µg·L^−1^. The artificial receptors used in this particular biosensor may provide valuable alternative to monoclonal antibodies as nonspecific interactions between the test assay and the target protein were minimized when these receptors were used. Another advantage of this assay was that it did not require serum pre-treatment [84].

In another study, oligonucleotide-functionalized AuNps (also termed as AuNPs probes) were integrated into circulating-flow QCM during the development of piezoelectric genosensor for the detection of DENV serotype 2 [85]. The assay employed two different AuNps probes which were designated as “mass enhancer” and “sequence verifier” probes. The mass enhancer probe amplified the DENV genome which was then specifically identified by sequence verifier probe resulting in oscillatory frequency change and subsequently detected by QCM as a detection signal. This genosensor was capable of detecting up to 2 PFU·mL^−1^ of target analyte from dengue-infected sera. This method was quite useful since it does not require expensive equipment and is a label-free assay [85].

#### 5.2.3. Optical Biosensor

Over the past decades, the optical biosensor has gained a lot of attention due to its high selectivity and sensitivity. Moreover, it is a label-free assay and provides real-time detection. The basic principle of optical biosensor is often based on the adsorption of the target analyte to a selective biorecognition element immobilized on the sensor surface, which causes a change on transducer such as fluorescent signal and measured by a fluorescent detector. Two of the most well-known optical biosensor categories are optical fibers and surface plasmon resonance (SPR) surfaces [62,86].

Among the reported optical biosensor is a generic semi-disposable microfluidic biosensor developed to detect RNA of DENV serotype 3 [87]. In this biosensor, two different DNA probes were used; capture probe which was coupled to supermagnetic beads for the immobilization of target RNA and DNA reporter probe which was conjugated with dye encapsulated liposome for fluorescent detection. Upon interaction with target RNA, both probes hybridized with a target analyte and resulted in liposome-target-bead complex. This biorecognition event was detected by a fluorescence microscopy. The amount of liposomes captured was proportion to the concentration of target RNA and the system had a detection limit of 10 pmol·L^−1^ of target analyte [87]. In a similar type of study, a microfluidic biosensor has been developed for the serotype-specific detection of DENV. In contrast to previous studies [87], which detected only intact dye encapsulated liposome, in this study, both intact and lysed liposome were detected using fluorescence microscopy. This assay showed excellent sensitivity with detection limit of 0.125 nM and 50 pM for intact and lysed liposome detection systems, respectively. This microfluidic biosensor had a short assay time of only 20 min, which was attributed to the presence of dextran sulfate in the hybridization buffer. Moreover, the study showed that this biosensor was capable of detecting all DENV serotypes using four different serotype specific capture probes [88]. Another group of researchers had developed a serotype specific and sensitive genosensor coupled with isothermal nucleic acid sequence-based amplification (NASBA) to amplify the amount of target RNA [89]. The important features of this genosensor were that it was portable, inexpensive and easy to use. In this assay, reporter probe coupled with dye-entrapping liposomes assay hybridized with NASBA amplified generic sequence was added to viral RNA. For the generic DENV biosensor, one conserved capture probe was used while for serotype specific biosensor four different capture probes were employed in this study. The amount of liposomes detected in hybridized complex of capture probe-target-reporter probe was equivalent to the concentration of target analyte in the sample. The capture zones were quantified using reflectometer. The result showed that this genosensor could detect as low as 10 PFU·mL^−1^ of target RNA. However, specificity analysis obtained using serotype specific capture probes showed that the genosensor could discriminate all serotypes of dengue, except dengue serotype 3 [89].

Serological methods have also been applied in the optical biosensor for the detection of DENV. In this regard, a label-free real-time assay was developed for the detection of dengue specific IgM antibody by using surface plasmon resonance. In this study, the sensing element used was DENV antigen which was covalently bound on gold sensor chip to capture the target analyte IgM antibody from serum. Interestingly, this study employed two immunoassay techniques, direct immunoassay to detect dengue specific IgM antibody and indirect competitive inhibition to detect DENV during acute dengue phase. In a direct immunoassay, when antibody from sera was adsorbed on the sensor with immobilized DENV antigen, an increase in the resonance angle observed. In the case of an indirect immunoassay, dengue specific monoclonal antibody was first incubated with DENV antigen. Later, this antibody and antigen incubated solution was added on the sensor with immobilized DENV antigen, resulting in a decrease of resonance angle which indicated an inhibitory reaction of the immunoassay. This type of biosensor may be quite useful for the diagnosis of both acute and late dengue infection. However, sensitivity and specificity of the assay was not evaluated using real serum samples [90]. Chemiluminescent measurements are very useful due to their high sensitivity, less background signal, wide dynamic range and comparatively inexpensive instrumentation. One study utilized the combined advantage of chemiluminescent hybridized with the optical fiber sensor [28]. Similarly, this chemiluminescent optical fiber immunosensor (OFIS) was also developed to detect dengue specific IgM antibody in the clinical serum sample. It was demonstrated that OFIS provided excellent sensitivity as the detection limit was ten times lower than chemiluminescent and 100 times lower than colorimetric ELISA [28].

To date, the biosensor based assay for the diagnosis of dengue have been developed using different types of transduction mechanisms as well as targeting different analytes and infection stages. In general, the developed biosensors with these various transduction mechanisms are highly sensitive in comparison to conventional methods making it a promising new diagnostic kit for dengue (Table 4).

**Table 4:** Reported LODs and assay times for dengue.

Transduction mechanism	Analyte (target)	Limit of detection (LOD)	Assay time (min.)	Detection in real sample/spiked sample	Ref.
**Electrochemical**	RNA	0.01 μM	–	–	[77]
	SG	80 dilution Fold	–	serum	[63]
	SG	1:150	–	serum	[65]
	NS1	0.33 ngm·L^−1^	–	serum	[66]
	NS1	0.12 ngm·L^−1^		spiked serum	[55]
	SG	80-dilution fold	–	serum	[70]
	Viral particle	1 PFU·mL^−1^	50	–	[75]
	Viral particle	0.230 and 0.710 PFU·mL^−1^	40	spiked serum	[76]
	RNA	0.92 nM	–	–	[78]
	cDNA	9.55 × 10^−12^ M	–	serum	[80]
	cDNA	2.7 × 10^−12^ M	–	–	[81]
	NS1	0.09 µg·mL^−1^		–	[71]
**Piezoelectric**	NS1	0.740 µg·mL^−1^		serum	[83]
	Envelope protein	1.727 µg·mL^−1^		serum	[83]
	cDNA	2 PFU·mL^−1^	90	spiked blood	[85]
	NS1	1–10 µg·L^−1^	20–30	blood	[84]
	NS1	0.05 μg·mL^−1^	30–60	–	[11]
**optical**	RNA	10 pmol·L^−1^	–	–	[87]
	RNA	0.125·nM	20	–	[88]
	cDNA	100 PFU·mL^−1^	–	serum	[89]
	IgM antibody	1:10^6^ dilution		serum	[28]
	IgM antibody	12 pg/mm^2^	–	blood plasma	[12]

## 6. Conclusions

Among the existing conventional methods, virus isolation is of limited use as it is laborious, expensive and requires sophisticated lab equipment. Alternatively, reverse transcriptase PCR could be used for the detection of dengue viral RNA during early stage of infection as it is rapid and sensitive technique although it needs technical skills and developed laboratory.

Because of this, serological-based assays are currently the most popular and widely used as they are comparatively cheap, sensitive, rapid and have long shelf life reagents. Among these serological assays, dengue NS1 ELISA is perhaps a more useful alternative to PCR during the acute dengue phase while dengue specific IgM ELISA remains a good choice during the convalescent period. Although these conventional techniques are very sensitive, they suffer from several drawbacks such as time-consuming, not adaptable to real-time detection, require skilled personnel and bulky and expensive machine. On the other hand, RDTs for NS1 antigen and IgM antibody detection based on immunochromatography are a good alternative to ELISA-based assay since they are easy to use, require no equipment and have very short assay time. Although RDTs can provide opportunities for point-of-care testing, their lack of sensitivity is a major challenge.

The development of biosensor assays may overcome the limitations of ELISA and RDTs since biosensor technology has sensitivity similar or higher than ELISA and with possible portability and miniaturization like RDTs. In addition, this technology is easy-to-use, inexpensive (since it requires less reagent and low energy consumption), has continuous monitoring, and ability to measure the target analyte in complex matrices with minimal sample preparation. To date, various transducers and various target analytes have been used during the development of sensitive, rapid and/or quantitative DENV biosensors.

However, there is still a long way before commercialization of dengue biosensor could be materialized as some issues regarding clinical samples as well as the biosensor itself need to be resolved. The clinical samples may require additional preparation such as prior RNA extraction before it could be used in genosensor, making it difficult to adopt this type of biosensor as a point-of-care test. Meanwhile, integration of major biosensor components, including biosensing agent, sample pre-treatment, transducer, and detection method into a fully automated yet portable system is a crucial step towards the development of commercialized biosensor. For the diagnosis of dengue, biosensor could only become alternative commercial assay to conventional tests if it could offer rapidity and simplicity equal to RDTs while maintaining the specificity of ELISA.
